# A loss-of-function variant in canine *GLRA1* associates with a neurological disorder resembling human hyperekplexia

**DOI:** 10.1007/s00439-023-02571-z

**Published:** 2023-05-24

**Authors:** Tiina Heinonen, Thomas Flegel, Hanna Müller, Alexandra Kehl, Sruthi Hundi, Kaspar Matiasek, Andrea Fischer, Jonas Donner, Oliver P. Forman, Hannes Lohi, Marjo K. Hytönen

**Affiliations:** 1 Department of Medical and Clinical Genetics, University of Helsinki, Helsinki, Finland; 2 Folkhälsan Research Center, Helsinki, Finland; 3 Department of Veterinary Biosciences, University of Helsinki, Helsinki, Finland; 4 Department of Small Animals, Leipzig University, Leipzig, Germany; 5 Tieraerztliches Fachzentrum Muehlhausen Dr. Ortmann & Dr. Stief, Muehlhausen/Thueringen, Germany; 6 Laboklin GmbH&CO.KG, Bad Kissingen, Germany; 7 Section of Clinical and Comparative Neuropathology, Institute of Veterinary Pathology, Centre for Clinical Veterinary Medicine, LMU Munich, Munich, Germany; 8 Clinic of Small Animal Medicine, Centre for Clinical Veterinary Medicine, LMU Munich, Munich, Germany; 9 Wisdom Panel Research Team, Wisdom Panel, Kinship, Helsinki, Finland; 10 Wisdom Panel Research Team, Wisdom Panel, Kinship, Leicestershire, UK

## Abstract

Hereditary hyperekplexia is a rare neuronal disorder characterized by an exaggerated startle response to sudden tactile or acoustic stimuli. In this study, we present a Miniature Australian Shepherd family showing clinical signs, which have genetic and phenotypic similarities with human hereditary hyperekplexia: episodes of muscle stiffness that could occasionally be triggered by acoustic stimuli. Whole genome sequence data analysis of two affected dogs revealed a 36-bp deletion spanning the exon–intron boundary in the glycine receptor alpha 1 (*GLRA1*) gene. Further validation in pedigree samples and an additional cohort of 127 Miniature Australian Shepherds, 45 Miniature American Shepherds and 74 Australian Shepherds demonstrated complete segregation of the variant with the disease, according to an autosomal recessive inheritance pattern. The protein encoded by *GLRA1* is a subunit of the glycine receptor, which mediates postsynaptic inhibition in the brain stem and spinal cord. The canine *GLRA1* deletion is located in the signal peptide and is predicted to cause exon skipping and subsequent premature stop codon resulting in a significant defect in glycine signaling. Variants in *GLRA1* are known to cause hereditary hyperekplexia in humans; however, this is the first study to associate a variant in canine *GLRA1* with the disorder, establishing a spontaneous large animal disease model for the human condition.

**Supplementary Information:**

The online version contains supplementary material available at 10.1007/s00439-023-02571-z.

## Introduction

Hereditary hyperekplexia or startle disease is a rare neurological disease characterized by sudden and exaggerated startle response to unexpected sensory stimulation, such as by simple and intense tactile or auditory stimuli. This is followed by an episode of general body stiffening with unaltered consciousness. The excessive startle response in people can be one feature in many genetic neurodevelopmental disorders, like early-infantile epileptic encephalopathies and neuropsychiatric syndromes, or it can have an acquired cause. The isolated hereditary hyperekplexia is caused by defective inhibitory glycinergic neurotransmission and has been associated with genes encoding glycine receptor (GlyR) subunits alpha and beta (*GLRA1*, *GLRB*) and solute carrier family 6 member 5 (*SLC6A5*), of which the variants in glycine receptor alpha 1 (*GLRA1*) constitute the vast majority (80%) of cases. Nearly 80 variants have been described in *GLRA1* so far, causing the disease either through dominant or recessive inheritance (Zhan et al. 2021).

Spontaneous hereditary hyperekplexia has also been reported in dogs (Gill et al. 2011; Murphy et al. 2019), horses (Gundlach et al. 1993), and cattle (Charlier et al. 2008). Two variants, a 4.2 kb microdeletion and a 2 bp deletion in *SLC6A5,* have been associated with the disease in Irish Wolfhounds and Spanish Greyhounds, respectively (OMIA 001594–9615, variants 638 and 1080). Here, we present a family of Miniature Australian Shepherds with exaggerated startle reflexes and hypertonia and identify a likely pathogenic deletion in *GLRA1*.

## Results

Two 2-month-old Miniature Australian Shepherd dogs (one female, one male) were presented for a 2-week history of sudden rear limb stiffness and subsequent collapse (Online Resource 1; Online Resource 2). The male puppy was more severely affected than the female. Both dogs were the smallest out of a litter of seven dogs (4 females, 3 males), and altered rear limb control had been observed since birth.

Both dogs were normal on general physical examination. The neurological examination did not reveal any deficits besides a stiff stilted gait, which was more pronounced in the rear limbs. Occasionally, the male dog suddenly became rigid in all four limbs and fell over to the side. Those collapses could sometimes, but not consistently, be induced by clapping hands. Consciousness was maintained during those episodes, which lasted up to 10 s before the dog could get up again. During those episodes, the dogs experienced evident dyspnea, which dissolved completely after being able to walk again.

Further diagnostics were performed for the male dog only. Blood chemistry was unremarkable. Complete blood count performed at 6 weeks of age identified a lowered packed cell volume of 23.6%, which appears relatively low, but was considered to be normal at that age (reference range for that age: 23–33% (Rørtveit et al. 2015)). C-reactive protein was elevated in serum at 24.7 mg/l (reference range: 0.0–10.0). Tests for genetic defects of the *CLCN1* gene, known to cause myotonia congenita in other canine breeds, such as American Bulldog (Rodrigues et al. 2020), Australian Cattle dog (Finnigan et al. 2007), Jack Russel Terrier (Lobetti 2009), Labrador Retriever (Quitt et al. 2018), and Miniature Schnauzer (Bhalerao et al. 2002) were negative.

Electromyography performed under general anesthesia revealed mild spontaneous electric activity, including fibrillation potentials and positive sharp waves in most proximal appendicular muscles. The motor nerve conduction velocity of the right sciatic nerve was 50 m/s (normal for dogs up to 3 months of age: > 40 m/s). Awake electromyography identified mild myotonic discharges in appendicular muscles.

**Fig. 1:**
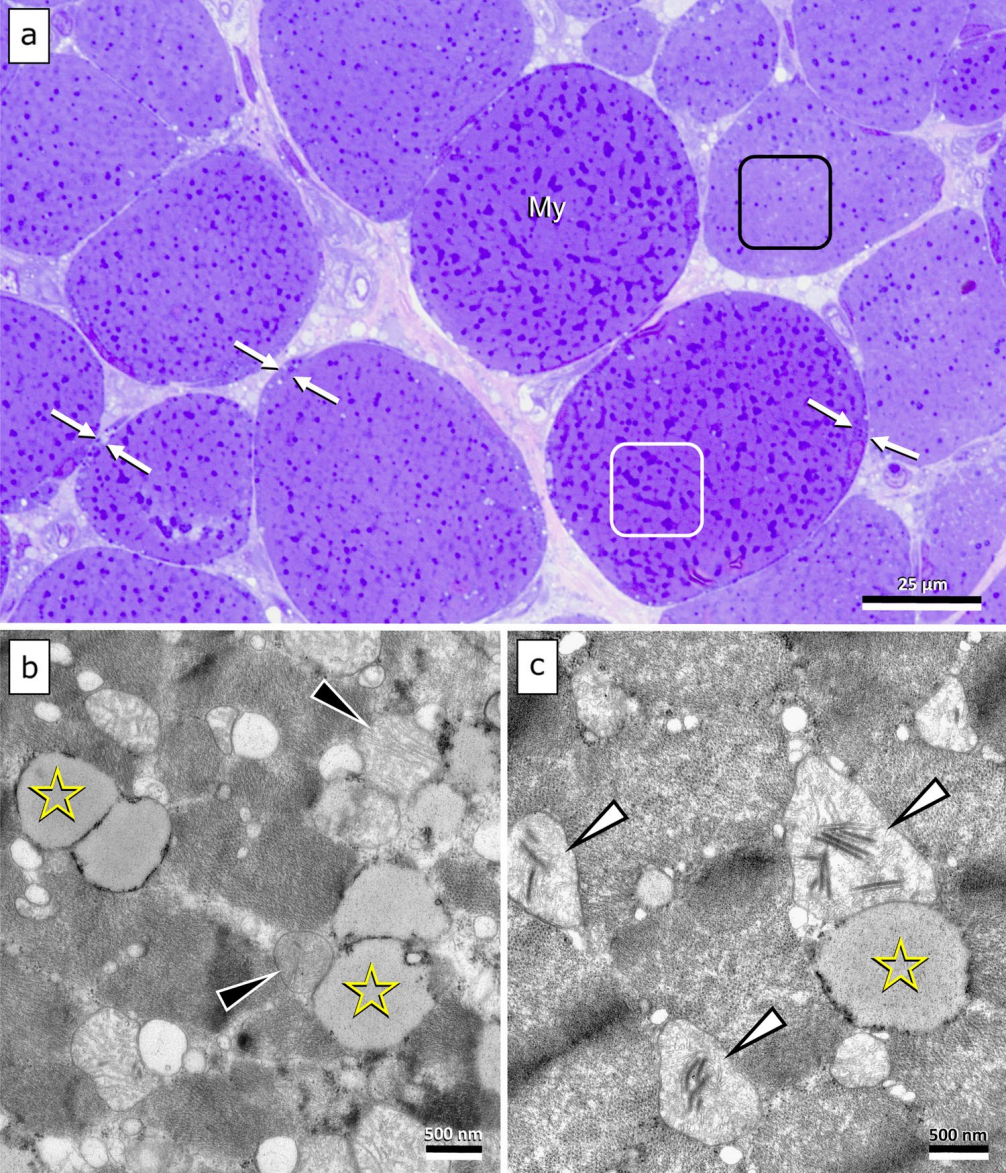
Most muscle fibers of both muscles showed a high load of partially confluent dark blue lipid droplets in between muscle filaments (**a**: white frame) and within the subsarcolemmal perinuclear cytoplasm (**a**: bound by arrows). At the ultrastructural level (**b, c**), the droplets present as homogenous semi electron dense conglomerates, typical for neutral fat (**b, c**: asterisks). They are located close to mitochondria (**a, b**: arrowheads), many showing disruption of cristae architecture and trilaminar inclusions (**b**: white arrowheads). Staining/contrasting: a: toluidine blue–safranin O; **b, c** uranyl acetate–lead citrate. Magnification: see scale bars

Morphological examination of the left semitendinosus and cranial tibial muscles revealed a moderate to marked increase of interfibrillar and subsarcolemmal lipid droplets going together with subhistological mitochondrial crowding and occasional cristae abnormalities (Fig. 1). Individual fibers at ultrastructural level also revealed I-band misalignment and early necrotic changes. There was no evidence of myonuclear abnormalities, fiber mineralization, sarcoplasmic vacuolation, protein inclusions, or pathological storage of polysaccharides. Those findings were interpreted as being consistent with a lipid storage myopathy.

Treatment using the following medications, one after the other, was attempted but failed to improve clinical signs: clonazepam 0.25 mg/kg TID per os, mexiletine 3.1 mg/kg TID per os, flecainide 3.1 mg/kg TID per os in addition to L-carnitine and vitamin Q10. Despite medication, there was a progression of clinical signs resulting in a complete inability to ambulate due to permanent muscle hypertonicity in all four limbs by the age of 3 months. In addition, the dog developed episodes of cyanosis during the most severe episodes of muscle stiffness, and it finally died at the age of 4 months.

The female littermate, which was affected but experienced a more protracted course of the disease, failed to improve on mexiletine as well and was euthanized at 7 months of age. The owner bred another litter of eight puppies (seven female and one male) with the same parents. The only male puppy of that litter developed the same clinical signs as the two dogs described before and died at 9 weeks, most likely due to respiratory failure. The affected puppies’ parents were not closely related: the inbreeding coefficient of the puppies was 0.00%, based on a five-generation pedigree with 18 missing relatives out of 62. DNA samples of all three affected puppies, as well as of both parents and five half-siblings, were collected for further genetic analysis.

### Whole genome sequencing analysis identifies a deletion in GLRA1

We performed whole genome sequencing of two affected dogs to identify the variant associated with the disease. We filtered the SNVs and indels according to an autosomal recessive disease using variant data of 598 dogs and 2 wolves as controls (Online Resource 3). The dogs and wolves used as controls in this gene discovery cohort had not shown clinical signs resembling hyperekplexia. The analysis revealed 1124 case-specific SNVs and indels. Of these variants, seven were in coding regions based on the Ensembl annotation, and four based on the NCBI annotation (Online Resource 4).

To prioritize the discovered exonic variants for further analyses, we performed an additional filtering with the same parameters, using variant data of 3649 dogs, 83 wolves and 4 coyotes as controls (Online Resource 3). In this cohort, phenotype data was not available for all samples. The analysis revealed 210 case-specific SNVs and indels, of which only 1 was in coding regions (g.4:58,338,953) when both the Ensembl and the NCBI annotations were considered (Online Resource 5). Additionally, we further investigated the seven coding region variants which remained after the initial gene discovery filtering (Online Resource 4). All except the variant in g.4:58,338,953 were observed in homozygous or heterozygous state in multiple breeds and were therefore excluded from further analyses.

**Fig. 2:**
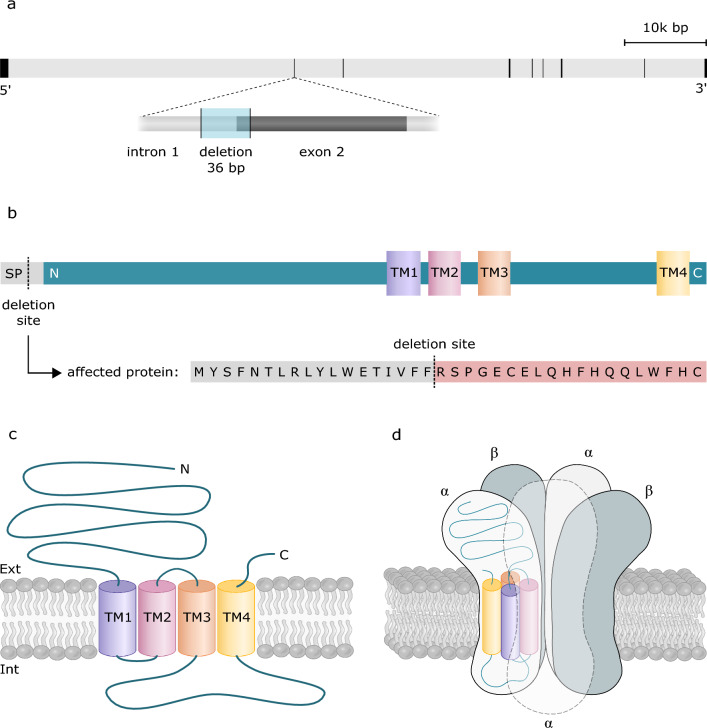
**a** The exon–intron structure of the *GLRA1* gene. The 36-bp deletion (position marked with dashed line) encompasses part of the intron 1 and exon 2. **b** *GLRA1* protein domains with the signal peptide (SP), N-terminus (N), four transmembrane domains (TM1–TM4) and C-terminus (C). The variant (highlighted with light blue) deletes a part encoding the signal peptide. The resulting protein is predicted to contain a part of the normal signal peptide followed by 19 abnormal amino acids (highlighted with red). **c** The structure of the glycine receptor alpha-1 subunit. **d** Heteromeric glycine receptors are formed by five subunits: three α subunits and two β subunits

The exonic variant in chromosome 4 (g.58,338,953) is a 36-bp deletion encompassing part of the intron 1 and exon 2 of glycine receptor alpha 1 (*GLRA1*) gene (Fig. 2a). The *GLRA1* gene encodes a glycine receptor subunit, which mediates postsynaptic inhibition in the central nervous system and the variants in the gene are known to cause hereditary hyperekplexia in humans (OMIM 138491, phenotype MIM number 149400). Therefore, we considered this variant to be an excellent candidate for the canine disease in this study.

### Variant validation demonstrates the allele segregation

**Fig. 3:**
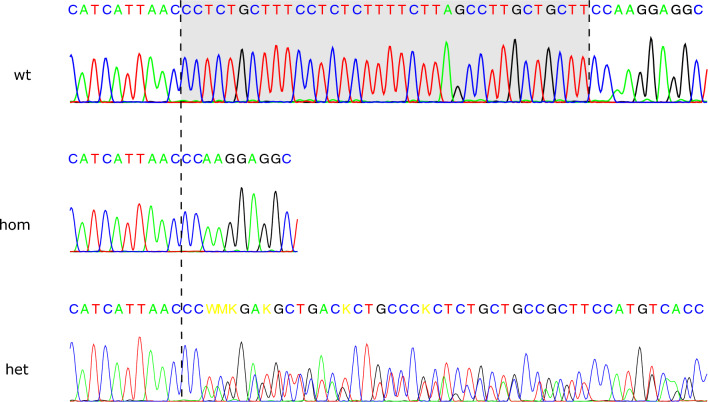
Sanger sequencing chromatograms for dogs homozygous for the reference allele (wt), affected individuals homozygous for the variant (hom), and heterozygous carriers (het). The deletion region is highlighted in gray in the reference allele in the uppermost image

We genotyped the pedigree samples (three affected siblings, their parents, and five healthy half-siblings) using Sanger sequencing to confirm the deletion and the allele segregation (Fig. 3). All three affected dogs were homozygous, and the parents were heterozygous for the alternative allele. In addition to this, four of the half-siblings were homozygous for the reference allele, and one half-sibling was a heterozygous carrier. The results indicate perfect segregation of the variant in the family according to a recessive disease.

To further validate the variant and investigate the allele frequency in the breed, we genotyped a cohort of 127 Miniature Australian Shepherds from Germany. This cohort was selected randomly from dogs whose samples had been submitted for commercial genetic testing. Of these, 121 dogs were observed to be homozygous for the reference allele, whereas 6 dogs were heterozygotes.

We also investigated the allele frequency in two breeds closely related to the Miniature Australian Shepherds: Miniature American Shepherds and Australian Shepherds. 45 Miniature American Shepherds and 74 Australian Shepherds from Finland were included in the screening cohort and genotyped for the variant. The Miniature American Shepherds and Miniature Australian Shepherds originate from the same population and are widely considered the same breed, with the difference that the Miniature American Shepherds are registered under the American Kennel Club (AKC) and Fédération Cynologique Internationale (FCI) kennel organizations, whereas the Miniature Australian Shepherds are not. Australian Shepherds were chosen to be screened since they are the most closely related breed to Miniature Australian and American Shepherd populations. All Miniature American Shepherds and Australian Shepherds were observed to be homozygous for the reference allele.

To investigate the allele frequency in other dog breeds, the *GLRA1* variant was further screened in a sample of 145,396 dogs of diverse breed ancestry submitted for commercial genetic testing. We identified 13 heterozygous dogs in the sample corresponding to a carrier frequency of 0.009%. All 13 carrier dogs were classified as mixed breed dogs and 12 of them originated from the USA. All carriers displayed an ancestry component from the Miniature American Shepherd breed ranging from 5.7 to 89.7% depending on the dog.

Even though the parents of the affected puppies were not closely related to each other based on their pedigrees, they both carried the rare *GLRA1* variant. To investigate the *GLRA1* haplotype background, we compared the SNVs of the two cases utilizing the WGS variant data and identified 14 intronic SNVs within the *GLRA1* gene. Both cases shared the same homozygous haplotype of 13 variants, located at g.4:58,336,657–58,353,369. The last intronic variant within *GLRA1* (g.4:58,384,457) was detected in a heterozygous state in one of the puppies. The haplotype background suggests that the defected *GLRA1* alleles of the parents are identical by descent inherited from a common ancestor. This was expected since the dog breeds are inbred populations.

The predicted open reading frame of canine *GLRA1* differs between Ensembl (ENSCAFT00805004856.1) and NCBI (XM_038535459.1), affecting the variant’s predicted effect on the protein (Online Resource 6). Alignment of the consequent protein sequences (ENSCAFP00805003700 and XP_038391387.1) to human GLRA1 (UniprotKB:P23415) demonstrated more similarity of the ENSCAFP00805003700 to human protein (Online Resource 6), most importantly at the translation initiation site, and therefore it was chosen for further *in silico* analyses. Human GLRA1 forms a pentameric receptor with glycine receptor beta-subunits (Grenningloh et al., 1990). The alpha 1 subunit contains a signal peptide, an extracellular domain that harbors the neurotransmitter binding site, and four α-helical transmembrane domains (Fig. 2b). The identified variant in *GLRA1* is predicted to delete 36 bp overlapping the exon–intron boundary, the region encoding the end of the signal peptide (Fig. 2b). Based on *in silico* analysis, there are no alternative splicing sites in exon 2 and therefore the deletion likely leads to skipping of exon 2 causing a frameshift and introduction of a premature stop codon. Likely, this leads to transcript degradation through nonsense-mediated decay before the transcript enters the translation machinery. If translated, the resulting protein is predicted to be significantly truncated, containing only part of the normal signal peptide, followed by 19 abnormal amino acids and, therefore, severely defective protein (Fig. 2b).

## Discussion

In this study, we describe episodic stiffness occasionally induced by acoustic stimuli in a family of Miniature Australian Shepherd dogs and associate it with a recessive truncating deletion variant in *GLRA1,* likely resulting in a complete knockout of the protein*.* The study establishes a natural large animal model for human *GLRA1*-related hereditary hyperekplexia.

Clinical signs in those affected Miniature Australian Shepherd dogs overlap but are not identical to hyperekplexia described in Irish Wolfhounds and Spanish Greyhounds (Gill et al. 2011; Murphy et al. 2019). The latter breeds started to develop clinical signs with the onset of ambulation, whereas the Miniature Australian Shepherd dogs were not affected before 6 weeks of age. In addition, Spanish Greyhounds and Irish Wolfhounds manifested permanent muscle stiffness exacerbated by movement and handling from the beginning on. In contrast, the Miniature Australian Shepherd dogs did initially experience only episodic generalized muscle hypertonicity, progressing to permanent hyperekplexia and incapacity to ambulate at 3 and 5 months of age.

Similarly, there are common features of hyperekplexia in humans and in the dogs described here, although there are also differences. Human hyperekplexia is characterized by generalized hypertonia and an exaggerated startle response that often can be induced by external stimuli (auditory, visual and tactile) starting early after birth (Zhan et al. 2021). However, there is some phenotypic variation of hyperekplexia, most likely caused by the large variety of genetic defects involving five genes related to the glycinergic neurotransmission system (Harvey et al. 2008; Bode and Lynch 2014; Zhan et al. 2021). Therefore, humans might only experience mild clinical signs with sporadic hypertonic jerks or may suffer from more severe clinical phenotypes with permanent generalized stiffness. Human clinical symptoms may improve within the first year of life but may also be persistent into adulthood.

Hereditary hyperekplexia is considered a treatable neurogenetic disorder in humans, with many patients responding favorably to clonazepam (Saini and Pandey 2020). The Miniature Australian Shepherds were presented with the same typical startle response induced by handling, excitement, and noises, but they did not start to do so before 6 weeks of age. In addition, clinical symptoms were progressive without responding to any of the medication given, including clonazepam, resulting in an inability to ambulate within a few weeks. However, it is difficult to compare clinical signs of one single variant in the canine family presented here with the wide spectrum of phenotypical expressions in humans, considering the large variety of genetic defects involving five different genes related to the glycinergic neurotransmission system in humans (Harvey et al. 2008; Bode and Lynch 2014; Zhan et al. 2021). The canine *GLRA1* variant described in this study is predicted to result in a loss of protein function and, consequently, likely to have a severe outcome. Therefore, as clonazepam treatment relieves the symptoms by enhancing the inhibitory effect of GABA, it might not be enough to rescue the inhibitory impact on canine patients. It is also worth noting that the variant identified in this study is located earlier in the *GLRA1* sequence than any reported variant in humans, being the only loss-of-function variant found in the sequence encoding the signal peptide. Thus, the affected dogs could contribute to understanding the genotype–phenotype correlation of different *GLRA1* variants.

Glycine receptors are formed by α and β subunits. *GLRA1* encodes the α_1_ subunit, one of the four known α subunit isoforms. Each subunit is constructed of a large extracellular amino terminus, four transmembrane domains connected by intracellular and extracellular loop structures, and a short extracellular carboxyl terminus (Fig. 2c). Glycine receptor is then formed by five subunits arranged around the central pore. The exact stoichiometry of heteromeric glycine receptors has been debated, but most evidence supports the alternating assembly of three α subunits and two β subunits (Fig. 2d) (Langosch et al. 1988; Kuhse et al. 1993; Burzomato et al. 2003; Patrizio et al. 2017). In addition to the heteromeric receptors, α_1_ and α_3_ subunits can form homomeric glycine receptors without any β subunits, whereas β subunits can not (Kuhse et al. 1993; Griffon 1999). However, heteromeric α_1_β receptors are probably the most common synaptic glycine receptors in adult humans. Due to the predominant role of α_1_ subunits in the synaptic glycine transmission and the inability of β subunits to form homomeric receptors, the lack of functional α_1_ subunits is likely to have a significant effect on mediating inhibitory neurotransmission. How the disinhibition may be related to the microscopical and ultrastructural muscle changes remains to be further explored. Lipid storage and mitochondrial abnormalities suggest a metabolic disturbance, such as defective β-oxidation (Pennisi et al. 2018), while muscle fiber necroses could also simply be a consequence of hyperrecruitment. However, excluding an incidental overlap syndrome requires investigation of further dogs affected by *GLRA1* mutation.

In addition to the Miniature Australian Shepherd samples, our validation cohort consisted of Miniature American Shepherds and Australian Shepherds. Including these breeds in the validation cohort was justified due to the close relationship between the three breeds. Miniature Australian Shepherds were derived from small-sized Australian Shepherds in the 1960s in the USA, yet the AKC recognized the breed not until 2015. On that occassion, the name was changed to Miniature American Shepherd, based on the breed’s origin. Although also the FCI approved the breed in 2019, some populations remained in small local registries with the breed name Miniature Australian Shepherd. Since these two populations have separated from each other very recently, they likely still are genetically indistinguishable. The presence of the *GLRA1* variant in the dogs with USA origin indicates that the allele is not restricted to the European population only. The commercial genetic testing will likely give more accurate information of the frequency and distribution of the allele within these breeds in the future.

The *GLRA1* haplotypes of the two affected puppies suggest that the parents likely have inherited the defected allele from a common ancestor. The genetic history of dog breed formation supports this theory. Modern dog breeds have been created via selective breeding with a limited population size, based on traits such as morphology and behavior. In addition, closed breed registries, use of popular sires and further selective breeding has resulted in progressively inbred populations. This has led to loss of genetic diversity within modern dog breeds. In Miniature American Shepherds, an average genetic inbreeding (*F*_adj_) of 0.104 has been reported (Bannasch et al. 2021)—as for comparison, the inbreeding coefficient for first-cousin offspring is 0.0625. Therefore, genetically, the parents of the affected puppies are probably more closely related than their 5-generation pedigrees suggest.

To summarize, this study describes a novel canine *GLRA1* variant causing clinical signs similar to human hereditary hyperekplexia. The results of this study will benefit both veterinary and human medicine. First, genetic testing will facilitate breeding decisions and thus help to prevent the production of affected dogs. In addition, this study establishes the affected dogs as large animal translational models for hereditary hyperekplexia.

## Materials and methods

### Study cohorts

EDTA blood and buccal mucosal samples were collected from 256 privately owned dogs, including 137 Miniature Australian Shepherds, 45 Miniature American Shepherds and 74 Australian Shepherds. The samples were stored at − 20 °C until genomic DNA was extracted using a semi-automated Chemagen extraction robot (PerkinElmer Chemagen Technologie GmbH). DNA concentration was determined either with NanoDrop ND-1000 UV/Vis Spectrophotometer, Qubit 3.0 Fluorometer (Thermo Fisher Scientific Inc.) or DeNovix DS-11 Spectrophotometer (DeNovix Inc., Wilmington, Delaware, USA).

### Clinical examination

General clinical examination and neurological examination were performed by a Diplomate of the European College of Veterinary neurology.

Electromyography was performed under general anesthesia and awake using the Neurowerk EMG and EEG recorder Morpheus (SIGMA Medizin Technik GmbH Neurowerk, Gelenau, Germany).

Genetic testing of known canine *CLCN1* variants (OMIA 000698-9615; variants 62, 609, 1041 and 1364) (Bhalerao et al. 2002; Finnigan et al. 2007; Lobetti 2009; Quitt et al. 2018; Rodrigues et al. 2020) was performed in Laboklin GmbH&CO.KG (Bad Kissingen, Germany).

### Histology

Biopsy samples were procured from the semitendinosus and tibialis cranialis muscle and shipped on cold packs overnight to the neuromuscular laboratory at the LMU Munich. Upon arrival, samples of each muscle were snap-frozen in isopentane cooled in liquid nitrogen, cryosectioned, and stained with hematoxylin–eosin, Engel’s modified Gomori trichrome stain, periodic acid Schiff and oil red O. Myofiber typing was performed via immunohistochemistry for myosin heavy chain. Additional sections underwent enzyme histochemistry for cytochrome oxidase and nicotinadmide adenine dinucleotide tetrazolium reductase. For evaluation of connective tissue components and blood vessels one piece of tissue per sample was immersed in 10% neutral buffered formalin to undergo paraffin sections, stained with hematoxylin–eosin and Masson’s trichrome stain. Furthermore, a set of muscle pieces was fixed with 6.25% glutaraldehyde and embedded in epoxy resin for semithin sections, stained with toluidine blue–safranin O. Areas abnormal on these sections were trimmed, sectioned at 50 nm thickness, and contrasted with uranyl acetate and lead citrate for transition electron microscopy. An experienced muscle pathologist carried out all these investigations.

### Whole genome sequencing

The affected Miniature Australian Shepherds were whole genome sequenced (WGS) using Illumina HiSeq X ultrahigh-throughput sequencing with paired-end reads (2 * 150 bp) at Novogene Beijing Institute. The latest canine genome assembly GSD1.0 (Wang et al. 2021), supplemented with the Y chromosome from ROS_Cfam_1.0, was used as the reference genome. The reads from WGS were aligned to the reference genome using BWA-MEM2 (Vasimuddin et al. 2019) and duplicate reads were marked with MarkDuplicatesSpark from Genome Analysis Tool Kit (GATK 4.2) (McKenna et al. 2010). Single nucleotide variants (SNVs) and indels were called using GATK 4.2 HaplotypeCaller in gVCF mode. The generated gVCF was combined with in-house sequenced samples gVCFs, generated in the same method, imported using genomicsDBImport from GATK 4.2, and genotyped together using GATK4.2 GenotypeGVCFs. Mobile Element Insertions were identified using Mobile Element Identifier Tool (MELT) (Gardner et al. 2017). Manta v.1.6 (Chen et al. 2016) software was used to identify structural variants (SVs), including insertions, deletions, inversions and duplications. The identified SVs were genotyped using Graphtyper2 (Eggertsson et al. 2019). Annovar (Wang et al. 2010) was used to annotate the SNVs, indels, SVs, and MEIs with functional annotation from NCBI and Ensembl.

### Variant analysis

For candidate variant filtering, the WGS data were imported to webGQT server, which provides a graphical interface for inheritance model-based variant filtering (Arumilli et al. 2020).

The filtering was performed in two stages. In the gene discovery stage, the variant data of two cases were filtered against 600 control genomes assuming an autosomal recessive inheritance. In the autosomal recessive model, the affected dogs were assumed to share the variants in a homozygous state. It was also assumed that the controls did not carry any copies of the variant, so no heterozygous calls were accepted per variant in the controls. Whole-exome sequencing and WGS data from 598 dogs from 96 breeds and two wolves (Online Resource 3) with 25X average depth (17–59X) were utilized for filtering the SNVs and indels, while 357 of these samples were available for MEI and SV analysis. The controls had been sequenced for our other ongoing projects and they had never shown clinical signs resembling hyperekplexia. Using the same parameters, we then prioritized the discovered exonic variants by filtering the variant data of the 2 cases against 3736 control genomes, including 3649 dogs, 83 wolves and 4 coyotes (Online Resource 3). These control genomes had been sequenced as a part of the Dog 10K genomes project (Ostrander et al. 2019). The variant data of all control genomes were aligned and called as described above. The publicly available variant data included in both filterings are listed in Online Resource 7.

Ensembl transcript ENSCAFT00805004856.1 and protein ENSCAFP00805003700 were used to count the nucleotide and amino acid positions for GLRA1, respectively. The protein alignment was performed with the Clustal Omega algorithm (https://www.ebi.ac.uk/Tools/msa/clustalo). The splice sites were predicted using the NNSPLICE 0.9 tool (Reese et al. 1997). *In silico* translation of the nucleotide sequence to protein sequence was performed with Translate tool on Expasy bioinformatics resource portal of the SIB Swiss Institute of Bioinformatics (Duvaud et al. 2021).

### Genomic DNA analysis

Genotyping of individual dogs was performed with PCR, followed by capillary sequencing. The DNA template containing the *GLRA1* variant position was amplified with Taq polymerase (Biotools B&M Labs, S.A.) using a forward primer (5′-GCAGCTTCTCCACAGCATGA-3′) and a reverse primer (5′-CATGTGTTTGCCATCTGCGT-3′) flanking the variant. The products were directly sequenced using the PCR primers on an ABI 3730 capillary sequencer (Life Technologies) after treatment with exonuclease I (New England Biolabs) and rapid alkaline phosphatase (Roche Diagnostics). The sequence data were analyzed using Unipro UGENE v1.32.0 (Okonechnikov et al. 2012; Golosova et al. 2014; Rose et al. 2019).

### Allele frequency in other dog breeds

The *GLRA1* gene variant was introduced into a custom-designed Illumina Infinium XT genotyping microarray (Illumina, Inc., San Diego, CA, USA), offered commercially for canine genetic disease panel screening purposes by Wisdom Panel (Portland, OR, USA). The *GLRA1* variant was screened in 145,396 dog samples voluntarily submitted for genetic testing with Wisdom Panel between October 2022 and January 2023. The breed ancestry of dogs was unconfirmed at the time of sample submission, and it was assigned based on comparison to a reference panel of over 21,000 dogs of known ancestry using the BCSYS Local Ancestry Classifier algorithm (Garrigan et al. 2021).

## Supplementary Information

Below is the link to the electronic supplementary material.

Online Resource 1. Video of an affected male puppy showing episodes of muscle stiffness (MP4 182799 KB)

Online Resource 2. Video of an affected female puppy showing episodes of muscle stiffness which can occasionally be triggered by acoustic stimuli (MP4 287149 KB)

Online Resource 3. The number of genomes and exomes from different breeds used as control variant data sets in filtering (XLSX 21 KB)

Online Resource 4. Gene discovery cohort: The case-specific SNVs and indels revealed by filtering the variant data assuming an autosomal recessive inheritance (XLSX 85 KB)

Online Resource 5. Variant prioritizing cohort: The case-specific SNVs and indels revealed by filtering the variant data assuming an autosomal recessive inheritance (XLSX 28 KB)

Online Resource 6. Alignment of the dog GLRA1 protein sequences (ENSCAFP00805003700 and XP_038391387.1) to human GLRA1 (UniprotKB:P23415) (PDF 66 KB)

Online Resource 7. List of the samples published in NCBI SRA (XLSX 89 KB)

## Data Availability

Whole genome sequencing data from the affected samples have been submitted to NCBI SRA (Bioproject-PRJNA828420 and SRA accession ids SRR18829224, SRR18829225).
