# Supplementary material for: A loss-of-function variant in canine GLRA1 associates with a neurological disorder resembling human hyperekplexia
Source: Hum Genet. 2023 May 24;142(8):1221–30. doi: 10.1007/s00439-023-02571-z (PMC10449970; doi:10.1007/s00439-023-02571-z)
Supplement: Supplementary file 6 — Online Resource 6. Alignment of the dog GLRA1 protein sequences (ENSCAFP00805003700 and XP_038391387.1) to human GLRA1 (UniprotKB:P23415) (PDF 66 KB) [file 439_2023_2571_MOESM6_ESM.pdf]

A loss-of-function variant in canine *GLRA1* associates with a neurological disorder resembling human hyperekplexia. Human Genetics.

Tiina Heinonen, Thomas Flegel, Hanna Müller, Alexandra Kehl, Sruthi Hundi, Kaspar Matiasek, Andrea Fischer, Jonas Donner, Oliver P Forman, Hannes Lohi*, Marjo K Hytönen*.

*Corresponding authors: Hannes Lohi (hannes.lohi@helsinki.fi) and Marjo K Hytönen (marjo.hytonen@helsinki.fi), Department of Medical and Clinical Genetics, University of Helsinki; Department of Veterinary Biosciences, University of Helsinki; Folkhälsan Research Center, Helsinki, Finland.

Alignment of the dog GLRA1 protein sequences (ENSCAFP00805003700 and XP_038391387.1) to human GLRA1 (UniprotKB:P23415) with the Clustal Omega algorithm.

```
XP_038391387.1            MALELVDLASGQTLVLSGEVCSGGAAFLSFLSLAASKEAEAARSAPKPMSPSDFLDKLMG   60
ENSCAFP00805003700        -------------MYSFNTLRLYLWETIVFFSLAASKEAEAARSAPKPMSPSDFLDKLMG   47
sp|P23415|GLRA1_HUMAN     -------------MYSFNTLRLYLWETIVFFSLAASKEAEAARSAPKPMSPSDFLDKLMG   47
                                       :   . :       : *:*****************************

XP_038391387.1            RTSGYDARIRPNFKGPPVNVSCNIFINSFGSIAETTMDYRVNIFLRQQWNDPRLAYNEYP   120
ENSCAFP00805003700        RTSGYDARIRPNFKGPPVNVSCNIFINSFGSIAETTMDYRVNIFLRQQWNDPRLAYNEYP   107
sp|P23415|GLRA1_HUMAN     RTSGYDARIRPNFKGPPVNVSCNIFINSFGSIAETTMDYRVNIFLRQQWNDPRLAYNEYP   107
                          ************************************************************

XP_038391387.1            DDSLDLDPSMLDSIWKPDLFFANEKGAHFHEITTDNKLLRISRNGNVLYSIRITLTLACP   180
ENSCAFP00805003700        DDSLDLDPSMLDSIWKPDLFFANEKGAHFHEITTDNKLLRISRNGNVLYSIRITLTLACP   167
sp|P23415|GLRA1_HUMAN     DDSLDLDPSMLDSIWKPDLFFANEKGAHFHEITTDNKLLRISRNGNVLYSIRITLTLACP   167
                          ************************************************************

XP_038391387.1            MDLKNFPMDVQTCIMQLESFGYTMNDLIFEWQEQGAVQVADGLTLPQFILKEEKDLRYCT   240
ENSCAFP00805003700        MDLKNFPMDVQTCIMQLESFGYTMNDLIFEWQEQGAVQVADGLTLPQFILKEEKDLRYCT   227
sp|P23415|GLRA1_HUMAN     MDLKNFPMDVQTCIMQLESFGYTMNDLIFEWQEQGAVQVADGLTLPQFILKEEKDLRYCT   227
                          ************************************************************

XP_038391387.1            KHYNTGKFTCIEARFHLERQMGYYLIQMYIPSLLIVILSWISFWINMDAAPARVGLGITT   300
ENSCAFP00805003700        KHYNTGKFTCIEARFHLERQMGYYLIQMYIPSLLIVILSWISFWINMDAAPARVGLGITT   287
sp|P23415|GLRA1_HUMAN     KHYNTGKFTCIEARFHLERQMGYYLIQMYIPSLLIVILSWISFWINMDAAPARVGLGITT   287
                          ************************************************************

XP_038391387.1            VLTMTTQSSGSRASLPKVSYVKAIDIWMAVCLLFVFSALLEYAAVNFVSRQHKELLRFRR   360
ENSCAFP00805003700        VLTMTTQSSGSRASLPKVSYVKAIDIWMAVCLLFVFSALLEYAAVNFVSRQHKELLRFRR   347
sp|P23415|GLRA1_HUMAN     VLTMTTQSSGSRASLPKVSYVKAIDIWMAVCLLFVFSALLEYAAVNFVSRQHKELLRFRR   347
                          ************************************************************

XP_038391387.1            KRRHHKSPMLNLFQEDEAGEGRFNFSAYGMGPACLQAKDGISVKGANNNNTTNPPPAPSK   420
ENSCAFP00805003700        KRRHHK--------EDEAGEGRFNFSAYGMGPACLQAKDGISVKGANNNNTTNPPPAPSK   399
sp|P23415|GLRA1_HUMAN     KRRHHKSPMLNLFQEDEAGEGRFNFSAYGMGPACLQAKDGISVKGANNSNTTNPPPAPSK   407
                          ******        **********************************.***********

XP_038391387.1            SPEEMRKLFIQRAKKIDKISRIGFPMAFLIFNMFYWIIYKIVRREDVHNQ             470
ENSCAFP00805003700        SPEEMRKLFIQRAKKIDKISRIGFPMAFLIFNMFYWIIYKIVRREDVHNQ             449
sp|P23415|GLRA1_HUMAN     SPEEMRKLFIQRAKKIDKISRIGFPMAFLIFNMFYWIIYKIVRREDVHNQ             457
                          **************************************************
```
